# Misleading menorrhagia in a peri-menopausal woman with underlying bowel cancer: a case report

**DOI:** 10.1186/1757-1626-1-36

**Published:** 2008-07-15

**Authors:** Hooman Soleymani majd, Lamiese Ismail, Sujit Datta, Sunil Doshi

**Affiliations:** 1 Milton Keynes General Hospital, Department of Obstetrics & Gynaecology, Standing Way, Eaglestone, Milton Keynes, MK6 5LD, UK; 2 Homerton University Hospital, Department of Obstetrics & Gynaecology, Homerton Row, Hackney, London, E9 6SR, UK; 3 Kettering General Hospital, Department of Obstetrics & Gynaecology, Rothwell Road, Kettering, Nothamptonshire, NN16 8UZ, UK

## Abstract

A peri-menopausal woman presented with symptoms and signs suggestive of fibroids. She was fit and healthy with no significant past medical history. She consented to having a hysterectomy but her surgery was performed prior to any diagnostic imaging being done.

At surgery there was an unexpected finding of disseminated carcinoma, diffusely infiltrating the uterus, fallopian tubes and ovaries. There was an omental cake that was biopsied. Frozen section showed signet ring cells, suggesting bowel carcinoma. Further intra-operative examination revealed a caecal tumour. After surgery she was investigated further and eventually referred for palliation, due to her advanced disease.

## Case presentation

A 46-year-old Caucasian woman presented to the gynaecology outpatient's department complaining of menorrhagia, bladder pressure and a feeling of heaviness in the pelvis. She had recently also started experiencing deep dyspareunia. She reported no bowel or constitutional symptoms.

She was a housewife and was a teetotal, non-smoker. She had one child, born by vaginal delivery and her previous cervical smears were normal. She was healthy and had no significant medical, surgical or family history. Examination revealed an enlarged, fourteen-week size uterus. There were no adnexal masses felt. In view of her symptoms, she was counselled about the various treatment options for menorrhagia. She declined all conservative measures and was very keen on having a total abdominal hysterectomy. An ultrasound scan was requested and her operation was booked.

Before diagnostic imaging could be performed, she attended hospital for her hysterectomy. Surgery was done through a Pfannenstiel incision, which revealed some haemorrhagic fluid in the abdomen. This was sent for cytology. The uterus was found to be uniformally enlarged, the fallopian tubes looked thickened and the ovaries appeared to have carcinomatous deposits on their surface.

The upper abdomen was explored. The omentum was caked with tumour, a biopsy was taken and frozen section performed. This showed deposits of mucin with some signet ring cells – suspicious of carcinoma of the bowel. The surgical consultant was called, at which time examination demonstrated a palpable tumour in the caecum. A decision was taken to proceed with total abdominal hysterectomy and bilateral salpingo-oophorectomy and to defer hemi-colectomy to a later date, until further investigations could be performed.

Subsequent gastroscopy was normal but her colonoscopy was limited due to looping of the bowel. A computerized tomography scan of the chest, abdomen and pelvis revealed no mediastinal nodes or lung parenchymal deposits. Her upper abdomen was normal. However there was thickening of the right para-colic area and caecal pole consistent with local infiltration.

Histology of her pelvic organs showed extensive infiltration of the cervix, basal endometrium, myometrium, ovaries and tubal tissues by poorly differentiated adenocarcinoma, with a signet ring pattern showing mucin production. This was identical to the omental biopsy. The impression was that these appeared to be metastases rather than intrinsic lesions.

The patient was later referred for palliative chemotherapy as the tumour was felt to be incurable. She subsequently died of her disease some twelve months later.

## Discussion

Bowel cancers invade adjacent structures dependent on their site. Ascending colon tumours tend to invade the duodenum and liver, whilst rectal tumours are more likely to invade the genital organs [1]. In general, non genital malignancies rarely metastasize into the uterus. But should this occur, metastatic uterine cancer may cause uterine enlargement and thickening of the myometrium [2]. Thus mimicking a variety of gynaecological symptoms, which could suggest intrinsic uterine pathology.

Our case report re-iterates the importance of considering bowel pathology in the differential diagnosis of all patients presenting with gynaecological symptoms. These days, it is vitally important to bear in mind that diagnostic imaging must be obtained prior to any surgical intervention, as we run the risk of missing serious pathology is we omit this step.

In this case imaging prior to surgery may not have changed the eventual outcome, but it may have triggered further investigation, which could have led to the correct diagnosis prior to surgery.

## Consent

Written informed consent was obtained from the patient's next of kin for publication of this case report. Unfortunately the patient has died as a result of her condition. A copy of the written consent is available for review by the Editor-in-Chief of this journal.

## Competing interests

The authors declare that they have no competing interests.

## Authors' contributions

Both SD's and HS were integrally involved in the patient's management and diagnosis. All also contributed to writing of the case report. LI performed the literature search and final copy-editing the paper. All authors read and approved the final manuscript.
